# High prevalence of sexually transmitted infections and risk factors among HIV-positive individuals in Yunnan, China

**DOI:** 10.1186/s40001-022-00635-w

**Published:** 2022-01-13

**Authors:** Wei Tu, Yu-Ye Li, Yi-Qun Kuang, Rong-Hui Xie, Xing-Qi Dong, Dan Zhang, Yan-Ling Ma, Wan-Yue Zhang, Lin Lu

**Affiliations:** 1 Department of Public Health, Kunming Medical University, Kunming, 650500 China; 2 Department of Dermatology and Venereology, First Affiliated Hospital of Kunming Medical University, Kunming, 650032 China; 3 NHC Key Laboratory of Drug Addiction Medicine, First Affiliated Hospital of Kunming Medical University, Kunming Medical University, Kunming, 650032 China; 4 Yunnan Provincial Hospital of Infectious Diseases/Yunnan AIDS Care Center (YNACC), Anning, 650300 China; 5 Yunnan Centers for Disease Control and Prevention, Kunming, 650022 China

**Keywords:** HIV, Syphilis, CT, NG, MG, HSV-2, Risk factor

## Abstract

**Background:**

Yunnan has the highest rates of HIV in China. Other treatable sexually transmitted infections (STIs) are associated with accelerated HIV transmission and poor ART outcomes, but are only diagnosed by syndromic algorithms.

**Methods:**

We recruited 406 HIV-positive participants for a cross-sectional study (204 ART-naive and 202 receiving ART). Blood samples and first-voided urine samples were collected. Real-time polymerase chain reaction methods were used for diagnosing *Chlamydia trachomatis* (CT), *Neisseria gonorrhea* (NG) and *Mycoplasma genitalium* (MG)*.* Syphilis and herpes simplex virus type 2 (HSV-2) tests were also performed.

**Results:**

Among the 406 participants, the overall prevalence of STIs was 47.0% and 45.1% in ART-naive individuals and 49.0% in individuals receiving ART, respectively. The testing frequencies were 11.6% (11.8% vs. 11.4%), 33.2% (29.4% vs. 37.1%), 3.2% (3.4% vs. 3.0%), 2.0% (3.4% vs. 0.5%) and 4.7% (6.4% vs. 3.0%) for active syphilis, HSV-2, CT, NG and MG, respectively. The percentage of multiple infections in both groups was 10.8% (22/204) in ART-naive participants and 9.9% (20/202) in participants receiving ART. Female sex, an age between 18 and 35 years, ever injecting drugs, homosexual or bisexual status, HIV/HBV coinfection, and not receiving ART were identified as risk factors. Self-reported asymptomatic patients were not eliminated from having a laboratory-diagnosed STI.

**Conclusions:**

The STI prevalence was 47.0% (45.1% vs. 49.0%), and HSV-2, syphilis and MG were the most common STIs in HIV-infected individuals. We found a high prevalence (6.4%) of MG in ART-naive individuals. HIV-positive individuals tend to neglect or hide their genital tract discomfort; thus, we suggest strengthening STI joint screening and treatment services among HIV-infected individuals regardless of whether they describe genital tract discomfort.

## Introduction

Sexually transmitted infections (STIs) present an ongoing and persistent global public health challenge. The latest World Health Organization (WHO) report estimates that among people aged between 15 and 49 years, there are 374.3 million new curable STI cases every year of either *Chlamydia trachomatis* (CT), *Neisseria gonorrhea* (NG), *Trichomonas vaginalis* (TV), or syphilis [1]. This means that STIs are responsible for more than one million new infections per day [1]. Evidence shows that genital ulcers caused by STIs are an important clinical manifestation that can increase the risk of human immunodeficiency virus type 1 (HIV-1) acquisition and replication [2].

The synergistic effect between STIs and HIV can destroy the health of HIV-infected individuals in various ways. First, some STIs generally destroy mucosal barriers, induce genital inflammation and ulceration, increase viral shedding of HIV in the genital tract, and increase the scale of susceptible immune cells [e.g., CD4^+^ T cells and dendritic cells (DCs)] in the genital tract, which may facilitate HIV transmission to sex partners by increasing HIV target cells [3–5]. Second, STIs can also inhibit the immune responses of skin-resident DCs and create a microenvironment in the host that is conducive to HIV infection; STIs not only help themselves but also create a “yellow brick road” for HIV-1, which is transmitted mostly through the sexual route [6, 7]. Third, STI–HIV coinfection can usually reduce the CD4^+^ T cell count and enhance the HIV viral load in blood plasma and genital secretions, which might decrease the effectiveness of antiretroviral therapy (ART) [8, 9]. Finally, STI–HIV coinfection is significantly associated with persistent immune activation and poorer CD4^+^ T cell recovery [10]. HIV can also accelerate the progression of other STIs. When immune function is compromised, STI–HIV coinfection is more difficult to treat and may prolong the course of the disease [11].

Yunnan Province had the earliest epidemic of AIDS in China and was the most heavily affected area, and it has the largest number of HIV-positive individuals. Along with the rapid development of the economy, HIV prevalence has expanded from high-risk groups to the general population, and sexual transmission has now replaced unsafe intravenous drug use (IDU) as the dominant route of HIV transmission in China [12]. Epidemiological studies show that STIs are a public health problem in this area. Among miners in Yunnan Province, the prevalence of CT, NG, syphilis and herpes simplex virus type 2 (HSV-2) has been reported to be 4.8%, 0.8%, 1.8% and 9.6%, respectively [13]. Among female sex workers involved in high-risk sexual behaviors, the prevalence of CT, NG, syphilis, HSV-2 and TV was 25.9%, 8.3%, 7.5%, 68.1% and 10.6%, respectively [14]. STIs such as CT, *Mycoplasma genitalium* (MG), syphilis and TV cause asymptomatic disease [15–18]. Hence, it is difficult to screen for and treat asymptomatic STIs, which can also induce genital inflammation and facilitate HIV transmission. Since HIV and STIs have almost the same routes of transmission and risk behaviors, we should develop a strategy to control the spread of these infections combined with HIV. However, limited studies have been conducted to determine the prevalence and risk factors for STI–HIV coinfection among HIV-positive participants in Yunnan, China. This study aimed to evaluate the prevalence of STI coinfection (syphilis, HSV-2, CT, NG and MG) and the risk factors for coinfection among HIV-positive participants in Yunnan Province.

## Methods

### Study design and population

The study was conducted in Yunnan Provincial Hospital of Infectious Diseases, a public medical facility in Yunnan, China. It offers services such as voluntary counseling and testing for HIV, free ART, and psychotherapy for AIDS patients. Our target population was HIV-positive individuals who are looking for ART services in this institution. We used cluster sampling to recruit our participants in this cross-sectional study. According to the general recruitment process, trained interviewers conducted a face-to-face interview with each participant using a structured questionnaire. Measures included demographic characteristics, drug use, and sexual behaviors. Laboratory technicians collected approximately 5 ml of venous blood using an anticoagulant tube, which was centrifuged immediately for 15 min to separate the serum. Finally, the participants provided a first-void urine specimen for the examination of CT, NG, and MG.

The cross-sectional study was conducted from September 2020 to June 2021 and included ART-naive participants and HIV-positive participants receiving ART who lived in Yunnan, China. A total of 406 HIV-positive participants (204 in the ART-naive group and 202 in the ART group) were recruited. Demographic data, including age, sex, nation, occupation, region, current marital status, education status, ever injecting drugs, sexual orientation, the number of sexual partners, condom use, HBV positivity, HCV positivity, routine blood tests, blood biochemistry, latest CD4^+^ T cell count and ART, were collected from the outpatient service system database and the questionnaire survey in Yunnan AIDS Care Center.

### Inclusion and exclusion criteria

The inclusion criteria were as follows: (1) patients diagnosed with HIV/AIDS; (2) male or female patients, aged ≥ 18 years; and (3) individuals who provided informed consent to participate in this study. The exclusion criteria were as follows: (1) patients known to be pregnant or lactating; (2) patients with serious liver, kidney, heart, brain and other dysfunctions or serious complications such as hypertension, diabetes, and coronary heart disease; (3) patients participating in other clinical studies; and (4) patients who used antimicrobials in the previous 15 days.

### Data collection and STI detection

Serum samples were tested for syphilis using Treponema pallidum particle agglutination (TPPA, Fujirebio Inc, Japan) and the toluidine red unheated serum test (TRUST, Shanghai Rongsheng Biotech Co. Ltd, China). Active syphilis-positive individuals were defined as those who were positive for both TPPA and TRUST. Serum samples were tested for HSV-2 immunoglobulin (IgG) antibodies using the HSV-2 IgG enzyme-linked immunoassay kit HerpesSelect 2 ELISA IgG (ACON Biotech Co. Ltd, China). First-void urine was collected from the participants for the detection of CT, NG and MG by polymerase chain reaction (PCR) using a CT RNA PCR kit, NG RNA PCR kit, and MG RNA PCR kit (Shanghai Rendu Biotech Co. Ltd, China). If any test appeared positive, the participants were notified and received treatment.

### Ethics approval and consent to participate

This study was approved by the ethics review boards of Kunming Medical University. All study participants were anonymous, and written informed consent was obtained. All experiments were performed per the approved guidelines and regulations according to the principles expressed in the Declaration of Helsinki, and the experimental protocols were approved by the review board of Yunnan Provincial Hospital of Infectious Diseases (No. 202114).

### Statistical analyses

The Chi-squared test or Fisher’s exact test for categorical variables and the *t* test for continuous variables were used to analyze differences in demographic characteristics and selected variables between the ART-naive group and ART group. Odds ratios (ORs) and their 95% confidence intervals (CIs) were calculated by using logistic regression analyses to evaluate the association between STI–HIV coinfection and its related factors with age, sex, nation, occupation, region, current marital status, education status, sexual orientation, ever injecting drugs, the number of sexual partners, condom use, HBV positivity, HCV positivity, latest CD4 count and ART. All statistical analyses were performed using SPSS 19 software (IBM Company, New York, USA).

## Results

### Characteristics of the participants

A total of 406 HIV-infected participants agreed to participate in the study, among whom 204 (50.25%) were ART-naive participants and 202 (49.75%) were ART participants. The characteristics of the ART-naive participants and participants receiving ART are summarized in Table 1. Compared to the participants receiving ART, the ART-naive participants had a significantly higher unemployment rate (16.2% versus 4%) and were more likely to be unmarried or single (68.1% versus 51%). A higher proportion of ever injecting drugs was found in the participants receiving ART (14.9%) than in the ART-naive participants (5.9%). Regarding sexual behaviors, compared to 2 (1%) of the participants receiving ART, a total of 63 (30.9%) ART-naive participants had multiple sexual partners in the past year. The mean number of sexual partners in the former group was 3.2 (± 0.2) compared to 1 (± 0.3) in the latter group. A higher proportion of never using condoms was found in the participants receiving ART (12.7%) than in the ART-naive participants (0%). The prevalence of genital tract discomfort was only 0.5% (2/406), and most of the participants remained silent or self-reported as asymptomatic.

**Table 1:** Characteristics of ART-naive and ART HIV^+^ participants

Characteristics	ART-naive (*n* = 204)	ART (*n* = 202)	*p* value
	*n* (%)	*n* (%)	
General			
Age, years, median (range)	37 (18–86)	39.5 (23–69)	
18–35	93 (45.6)	74 (36.6)	
≥ 36	111 (54.4)	128 (63.4)	0.067
Sex			
Male	167 (81.9)	158 (78.2)	
Female	37 (18.1)	44 (21.8)	0.358
Nation			
Ethnic Han	171 (83.8)	179 (88.6)	
Other	33 (162)	23 (11.4)	0.162
Occupation			
Employee	121 (59.3)	115 (56.9)	
None	33 (16.2)	8 (4.0)	
Unknown	50 (24.5)	79 (39.1)	0.001
Region			
Yunnan	192 (94.1)	194 (96.0)	
Other province	12 (5.9)	8 (4.0)	0.371
Current marital status			
Married	65 (31.9)	99 (49.0)	
Unmarried or single	139 (68.1)	103 (51.0)	< 0.001
Education			
Up to primary	120 (58.8)	114 (56.4)	
Above primary	84 (41.2)	88 (43.6)	0.626
Sexual orientation			
Heterosexual	125 (61.3)	135 (66.8)	
Homosexual/bisexual	79 (38.7)	67 (33.2)	0.243
Ever injected drugs			
Yes	12 (5.9)	30 (14.9)	
No	192 (94.1)	172 (85.1)	0.003
No. of sexual partners			
Single	58 (28.4)	53 (26.2)	
Multiple	63 (30.9)	2 (1.0)	
Unknown	83 (40.7)	147 (72.8)	< 0.001
Condom use			
Consistent/casual	121 (59.3)	79 (39.1)	
Never	26 (12.7)	0 (0.0)	
Unknown	57 (28.0)	123 (60.9)	< 0.001
Genital tract discomfort			
Yes	2 (1.0)	0 (0)	
No or unanswered	202 (99.0)	202 (100.0)	0.483
CD4, median (range)	273 (1–1148)	731 (551–2200)	
Normal	28 (13.7)	202 (100.0)	
Down	176 (86.3)	0 (0.0)	< 0.001
CD4/CD8, median (range)	0.25 (0.02–3.19)	0.88 (0.30–6.75)	
Normal	16 (7.8)	135 (66.8)	
Down	160 (78.4)	65 (32.2)	< 0.001
Total T lymphocytes, median (range)	1314 (112–5416)	1815 (923–3840)	
Normal	111 (54.4)	180 (89.1)	
Down	39 (19.1)	1 (0.5)	< 0.001

*P values were based on the Chi-square test (or Fisher exact test) for categorical variables*

The statistical significance was indicated as *p < 0.05, and **p < 0.001

Created by the authors

**Fig. 1:**
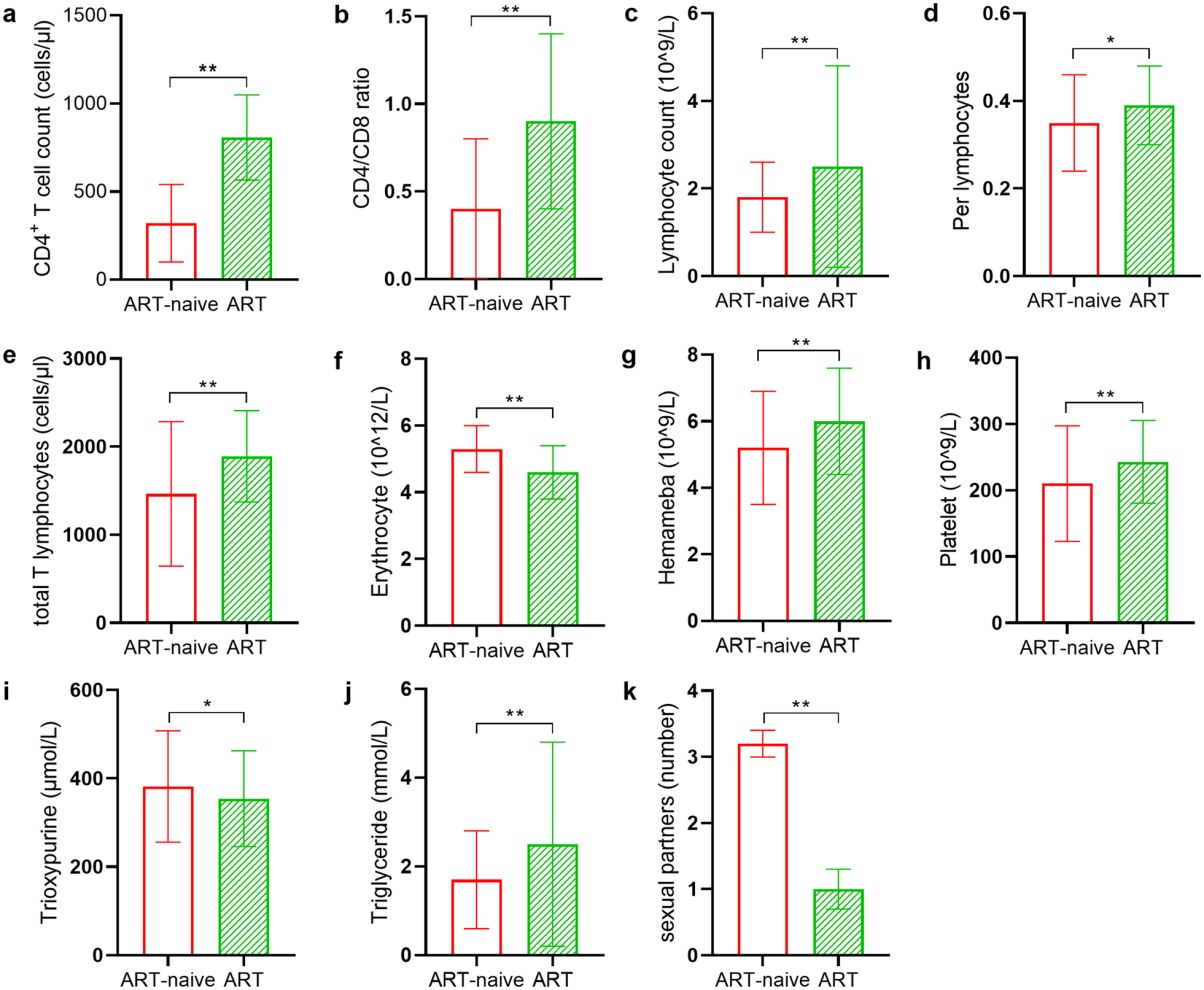
Comparison of laboratory data between the ART-naive participants and participants receiving ART. **a** CD4^+^ T cell count (cells/µl). **b** Ratio of CD4^+^T cell count/CD8^+^ T cell count. **c** Lymphocyte count (10^9^/L). **d** The percentage of lymphocytes (%). **e** Total T lymphocyte count (cells/µl). **f** Erythrocyte count (10^12^/L). **g** Hemameba count (10^9^/L). **h** Platelet count (10^9^/L). **i** Trioxypurine level (µmol/L). **j** Triglyceride level (mmol/L). **k** Number of sexual partners. The red bar shows the ART-naive group (*n* = 204), and the green bar shows the ART group (*n* = 202). Data are presented as the mean ± SEM. Statistical significance was indicated as *p < 0.05 and **p < 0.001. “Created by the authors”

The average count of CD4 + T cells in ART-naive participants was significantly lower than that in participants receiving ART (mean: 321 cells/µl versus 807 cells/µl). Similarly, the average count of total T lymphocytes was lower in the ART-naive participants than in the participants receiving ART (mean: 1464 cells/µl versus 1890 cells/µl). Therefore, the CD4/CD8 count was also decreased in ART-naive participants (0.4 versus 0.9). Compared with the ART-naive participants, the participants receiving ART had a higher average white blood cell count, percentage of lymphocytes, lymphocyte count, platelet count and triglyceride count, but a lower average erythrocyte count and trioxypurine level (Fig. 1). In addition, the age, sex, nation, region, education, sexual orientation and other data between the ART-naive and ART groups were not significantly different (*p* > 0.05).

### Coinfections and multiple infections in ART-naive participants and participants receiving ART

The prevalence of syphilis, HSV-2, CT, NG and MG among the participants was 19.9% (ART-naive: 19.6% and ART: 20.3%), 33.2% (ART-naive: 29.4% and ART: 37.1%), 3.2% (ART-naive: 3.4% and ART: 3.0%), 2.0% (ART-naive: 3.4% and ART: 0.5%), and 4.7% (ART-naive: 6.4% and ART: 3.0%), respectively (Table 2). The percentage of coinfections and multiple infections was 10.8% (22/204) in the ART-naive participants and 9.9% (20/202) in the participants receiving ART.

**Table 2:** Prevalence of sexually transmitted infections among HIV^+^ participants

Variable	Total (*n* = 406)	ART-naïve (*n* = 204)	ART (*n* = 202)
	Case number (%)	Case number (%)	Case number (%)
Syphilis			
TPPA +	81 (19.9)	40 (19.6)	41 (20.3)
TPPA + TRUST +	47 (11.6)	24 (11.8)	23 (11.4)
HSV-2 IgG +	135 (33.2)	60 (29.4)	75 (37.1)
*Neisseria gonorrhoeae*	8 (2.0)	7 (3.4)	1 (0.5)
*Chlamydia trachomatis*	13 (3.2)	7 (3.4)	6 (3.0)
*Mycoplasma genitalium*	19 (4.7)	13 (6.4)	6 (3.0)
STI	191 (47.0)	92 (45.1)	99 (49.0)

Created by the authors

**Fig. 2:**
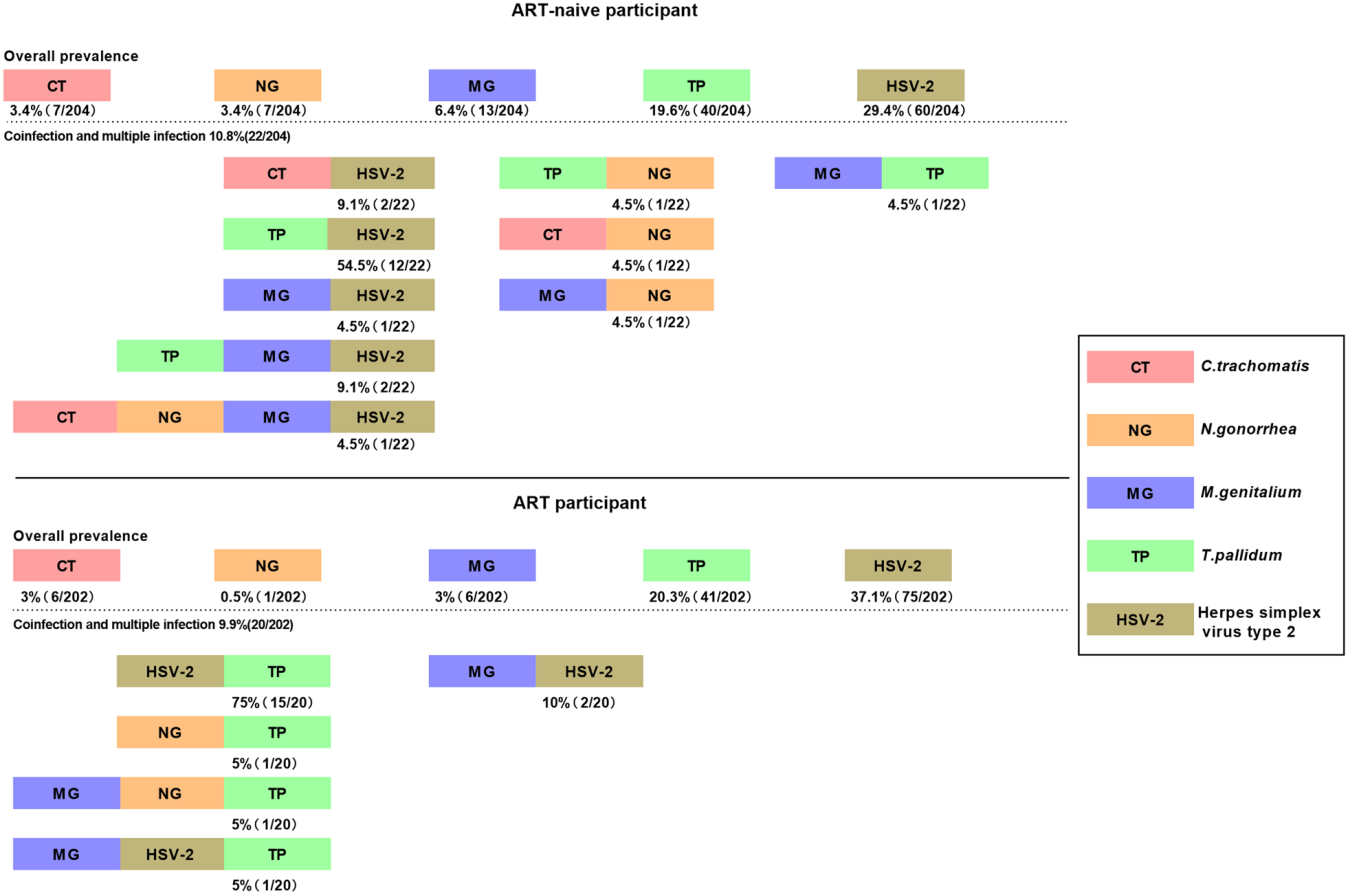
Prevalence of STIs among HIV-infected patients. The different-colored squares represent different STIs. HSV-2, TP, CT, NG and MG indicate herpes simplex virus type 2, *T. pallidum, C. trachomatis, N. gonorrhea* and *M. genitalium*, respectively. “Created by the authors”

For the ART-naive participants, the coinfection rate by decreasing order of prevalence was 54.5% (12/22) for *Treponema pallidum* (TP) with HSV-2, 9.1% (2/22) for CT–HSV-2, 9.1% (2/22) for TP–MG–HSV-2 and 4.5% (1/22) for MG–HSV-2, TP–NG, CT–NG, MG–NG, MG–TP, and CT–NG–MG–HSV-2. The participants in the ART group had a lower diversity of STIs. The coinfections by decreasing order of prevalence were 75.0% (15/20) for TP–HSV-2, 10.0% (2/20) for MG–HSV-2, and 5% (1/20) for TP–NG, TP–NG–MG and TP–MG–HSV-2 (Fig. 2).

### Factors associated with STI–HIV coinfection

All of the items between the STI–HIV coinfection groups were included in the logistic regression model (Table 3). For all participants, the logistic regression analyses revealed that female sex was significantly associated with STI–HIV coinfection risk (OR = 3.46, 95% CI 1.95–6.15, *p* < 0.001). In the further stratification analysis of the ART-naive and ART groups, female sex also showed consistent significant associations with STI–HIV coinfection risk (OR = 3.89, 95% CI 1.59–9.50, *p* = 0.003 versus OR = 4.21, 95% CI 1.84–9.61, *p* = 0.001).

**Table 3:** Logistic regression analyses of related factors associated with curable STI–HIV coinfection among HIV^+^ participants

Variable	STI–HIV coinfection rate, (*N* = 406), *n* (%)			STI–HIV coinfection rate on ART-naive, (*N* = 204), *n* (%)			STI–HIV coinfection rate on ART,(*N* = 202), *n* (%)		
	n/N (%)	OR (95% CI)	*P value*	n/N (%)	OR (95% CI)	*P value*	n/N (%)	OR (95%CI)	*P value*
Age, years									
18–35	72/167 (43.1)	Ref.		37/93 (39.8)	Ref.		35/74 (47.3)	Ref.	
≥ 36	119/239 (49.8)	1.24 (0.70–2.19)	0.461	55/111 (49.5)	1.12 (0.48–2.60)	0.791	64/128 (50.0)	1.08 (0.45–2.62)	0.858
Sex									
Male	136/325 (41.8)	Ref.		66/167 (39.5)	Ref.		70/158 (44.3)	Ref.	
Female	55/81 (67.9)	3.46 (1.95–6.15)	< 0.001	26/37 (70.3)	3.89 (1.59–9.50)	0.003	29/44 (65.9)	4.21 (1.84–9.61)	0.001
Nation									
Ethnic Han	168/350 (48.0)	Ref.		79/171 (46.2)	Ref.		89/179 (49.7)	Ref.	
Other	23/56 (41.1)	0.66 (0.36–1.22)	0.186	13/33 (39.4)	0.60 (0.25–1.42)	0.245	10/23 (43.5)	0.61 (0.23–1.62)	0.324
Occupation									
Employee	110/236 (46.6)	Ref.		52/121 (42.9)	Ref.		58/115 (50.4)	Ref.	
None	21/41 (51.2)	1.26 (0.61–2.59)	0.528	16/33 (48.5)	1.06 (0.44–2.54)	0.896	5/8 (62.5)	2.70 (0.56–13.05)	0.216
Others	60/129 (46.5)	0.89 (0.55–1.42)	0.614	24/50 (48.0)	0.94 (0.44–2.01)	0.880	36/79 (45.6)	1.01 (0.52–1.99)	0.966
Region									
Other province	10/20 (50.0)	Ref.		5/12 (41.7)	Ref.		5/8 (62.5)	Ref.	
Yunnan	181/386 (46.9)	1.08 (0.43–2.73)	0.864	87/192 (45.3)	1.13 (0.31–4.21)	0.853	94/194 (48.4)	0.92 (0.21–4.09)	0.916
Current marital status									
Married	75/164 (45.7)	Ref.		30/65 (46.2)	Ref.		45/99 (45.4)	Ref.	
Unmarried/single	116/242 (47.9)	1.31 (0.79–2.18)	0.304	62/139 (44.6)	1.06 (0.49–2.26)	0.884	54/103 (52.4)	1.52 (0.71–3.25)	0.278
Education									
Up to primary	113/234 (48.3)	Ref.		58/120 (48.3)	Ref.		55/114 (48.2)	Ref.	
Above primary	78/172 (45.3)	1.13 (0.69–1.87)	0.622	34/84 (40.5)	1.03 (0.49–2.18)	0.932	44/88 (50.0)	1.32 (0.64–2.73)	0.461
Sexual orientation									
Heterosexual	125/260 (48.1)	Ref.		61/125 (48.8)	Ref.		64/135 (47.4)	Ref.	
Homosexual/bisexual	66/146 (45.2)	1.55 (0.88–2.72)	0.129	31/79 (39.2)	1.39 (0.62–3.15)	0.426	35/67 (52.2)	1.58 (0.67–3.76)	0.297
Ever injected drugs									
No	168/364 (46.1)	Ref.		85/192 (44.3)	Ref.		83/172 (48.3)	Ref.	
Yes	23/42 (54.8)	1.40 (0.64–3.08)	0.396	7/12 (58.3)	1.19 (0.22–6.59)	0.839	16/30 (53.3)	1.71 (0.67–4.38)	0.265
No. of sexual partners									
Single	53/111 (47.7)	Ref.		29/58 (50.0)	Ref.		24/53 (45.3)	Ref.	
Multiple	21/65 (32.3)	0.48 (0.23–1.00)	0.051	19/63 (30.2)	0.42 (0.18–0.98)	0.054	2/2 (100)		
Others	117/230 (50.9)	1.28 (0.66–2.46)	0.466	44/83 (53.0)	1.32 (0.51–3.41)	0.567	73/147 (49.7)	1.33 (0.48–3.72)	0.588
Condom use									
Consistent	55/118 (46.6)	Ref.		18/39 (46.2)	Ref.		37/79 (46.8)	Ref.	
Casual/never	47/108 (43.5)	1.27 (0.63–2.58)	0.508	47/108 (43.5)	0.90 (0.37–2.17)	0.815	0		
Others	89/180 (49.4)	0.85 (0.45–1.60)	0.607	27/57 (47.4)	0.49 (0.16–1.44)	0.193	62/123 (50.4)	1.31(0.54–3.17)	0.545
CD4 count									
≥ 550	112/230 (48.7)	Ref.		13/28 (46.4)	Ref.		99/202 (49.0)	Ref.	
< 550	79/176 (44.9)	0.90 (0.52–1.56)	0.714	79/176 (44.9)	0.93 (0.37–2.33)	0.872	0 (0.0)	NA	NA
HBV									
Negative	174/374 (46.5)	Ref.		77/182 (42.3)	Ref.		97/192 (50.5)	Ref.	
Positive	17/32 (53.1)	1.19 (0.55–2.57)	0.654	15/22 (68.2)	2.76 (1.00—7.67)	0.051	2/10 (20.0)	0.25 (0.05–1.18)	0.080
HCV									
Negative	177/381 (46.5)	Ref.		82/188 (43.6)	Ref.		95/193 (49.2)	Ref.	
Positive	14/25 (56.0)	1.04 (0.40–2.74)	0.934	10/16 (62.5)	1.50 (0.33–6.80)	0.598	4/9 (44.4)	0.58 (0.12–2.89)	0.501

Created by the authors

**Fig. 3:**
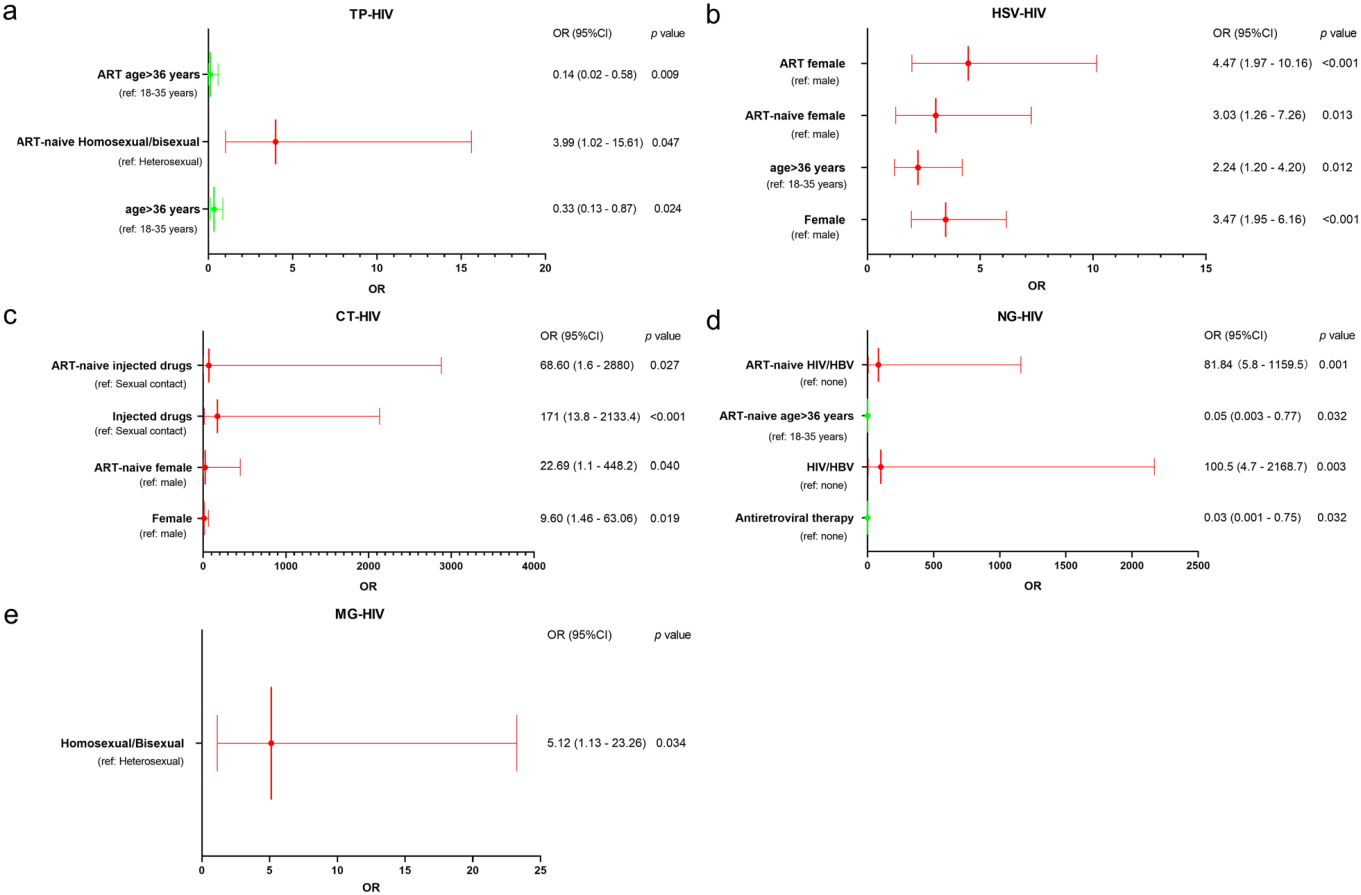
Factors associated with STIs among HIV-positive participants. **a** Factors associated with HIV and active syphilis coinfection. **b** Factors associated with HIV and HSV-2 coinfection. **c** Factors associated with HIV and CT coinfection. **d** Factors associated with HIV and NG coinfection. **e** Factors associated with HIV and MG coinfection. The red line shows the risk factors associated with each coinfection, the green line shows the protective factors, and the ref are the risk factors in each coinfection. *p < 0.05 (Logistic regression). “Created by the authors”

The identification of risk factors for each STI was determined by a logistic regression model. In the logistic regression analysis, all variables were adjusted, and the following variables remained in the model as risk factors: an age > 36 years (OR = 0.33, 95% CI 0.13–0.87, *p* = 0.024) reduced the risk of TP–HIV coinfection. An age > 36 years (OR = 2.24, 95% CI 1.20–4.20, *P* = 0.012) and female sex (OR = 3.47, 95% CI 1.95–6.16, *p* < 0.001) significantly increased the risk of an HIV–HSV-2 coinfection. Ever injecting drugs (OR = 171.00, 95% CI 13.80–2133.40, *P* < 0.001) and female sex (OR = 9.60, 95% CI 1.46–63.06, *P* = 0.019) strongly associated with CT–HIV coinfection risk. Coinfection with HBV (OR = 100.50, 95% CI 4.70–2168.70, *p* = 0.003) increased the risk of NG–HIV coinfection; however, receiving antiretroviral therapy (OR = 0.03, 95% CI 0.001–0.75, *p* = 0.032) became a protective factor against NG–HIV coinfection. We also found that homosexual or bisexual status (OR = 5.12, 95% CI 1.13–23.26, *p* = 0.034) was significantly associated with an increase in MG–HIV coinfection. The logistic regression analysis showed the following variables as risk factors for each STI coinfection among participants with ART-naive status: homosexual or bisexual status (OR = 3.99, 95% CI 1.02–15.61, *p* = 0.047) for TP–HIV coinfection, female sex (OR = 3.03, 95% CI 1.26–7.26, *p* = 0.013) for HIV–HSV-2 coinfection, ever injecting drugs (OR = 68.60, 95% CI 1.60–2880.00, *p* = 0.027) and female sex (OR = 22.69, 95% CI 1.10–448.20, *p* = 0.040) for CT–HIV coinfection, an age > 36 years (OR = 0.05, 95% CI 0.003–0.77, *p* = 0.032) and coinfection with HBV (OR = 81.84, 95% CI 5.80–1159.50, *p* = 0.001) for NG–HIV (Fig. 3).

## Discussion

This is the first study of STI prevalence that included a large number of new HIV-infected people and was detected by blood samples and first-voided urine samples in Yunnan, China. In the present study, a rather high STI prevalence among HIV-positive individuals was confirmed, namely, 47.0% (191/406) in total, 45.1% (91/204) in ART-naive individuals and 49.0% (99/202) in individuals receiving ART. This prevalence is much higher than the observed prevalence of 5.3% in HIV-positive individuals in Nepal [19], 11.1% in women living with HIV/AIDS (WLHA) in Uganda [20] and 23.5% in HIV-infected men who have sex with men (MSM) in China [21], but the prevalence was less than that of 60.6% in female sex workers in Peru [22] and 60.5% in HIV-infected women in Zimbabwe [23]. The prevalence of genital tract discomfort was 0.5% (2/406), which was lower than the STI prevalence. It was interesting to find that HIV-positive individuals tend to neglect or hide their genital tract discomfort. However, our health policy is that no symptoms means no screening, and these reasons may contribute to the high prevalence of STIs. In this study, we found that the overall prevalence of STIs among individuals receiving ART was slightly higher than that among ART-naive individuals, although the difference was not statistically significant. This may be associated with the high prevalence of HSV-2/HIV coinfection: 29.4% (60/204) in the ART-naive group versus 37.1% (75/202) in the ART group. In HIV-positive individuals, genital herpes tends to recurrently attack, and some individuals are even asymptomatic [24]. Without intervention, HSV-2 infection will persist, accumulate, and be transmitted to people living with HIV/AIDS. However, the ART-naive group had a higher diversity of STIs, coinfections and more sexual partners, and the prevalence of CT, NG and MG was also higher in this study. We believe that ART-naive individuals may face greater threats of STIs and remain in a more dangerous situation.

HSV-2 was the most common STI that was detected, with an overall prevalence of 33.2% (135/406) in HIV-positive individuals in this study. The prevalence of HSV-2/HIV coinfection was much higher than the 6%-13% observed in noninjecting drug users [25], but it was less than that of 48.6% in HIV-positive MSM in Shenyang [26]. Active syphilis was also a familiar STI that was observed, with a prevalence of 11.8% (24/204) in the ART-naive group and 11.4% (23/202) in the ART group. The result is similar to the prevalence of 11.3% that was detected in HIV-positive MSM in Zhejiang Province [27]; however, the prevalence of active syphilis detected in our study was less than that of 34.3% in HIV-positive MSM in Shenyang and 82.9% in HIV-positive MSM in the United States [26, 28]. HSV-2 and TP can induce genital ulceration, which plays an important role in facilitating the acquisition and transmission of HIV and other STIs. Evidence shows that 60.7% of HSV-2 infections and 3.9% of TP infections could be detected in ulcer specimens, and 68% of HSV-positive participants were coinfected with HIV in South Africa [29]. Moreover, among people with genital ulcers, the positivity rates of HSV, TP and HIV were 38.5%, 16.0% and 52.2%, respectively, in Zimbabwe [30].

The prevalence of CT that was detected in the two groups of participants (3.4% vs. 3.0%) was larger than that of 0.9% that was identified in WLHA [20], while it was smaller than that of 11.1% in MSM in Port-au-Prince [31]. The prevalence of NG identified in our study (3.4% vs. 0.5%) was lower than that of 5.4% that was observed in WLHA [20] and that of 7.2% in HIV-positive MSM in Birmingham [32].

Very few studies of MG prevalence have been assessed and generally associated with specific groups of women [33]. Our study provides the first report of MG prevalence among ART-naive individuals in Yunnan. We identified a prevalence of 6.4% (13/204) of MG infection in the ART-naive participants. This prevalence was higher than that of 2.4% that was detected in HIV-infected women in Brazil [34], but the prevalence was lower than that of 10.5% in HIV-positive women in Zimbabwe [35]. However, increasing evidence shows an association between MG and HIV infections. The report shows that the prevalence of MG was comparable to that for CT, NG and HSV among HIV-positive men, and MG infection may be associated with HIV shedding in the genital tract [35]. MG infection had a prevalence of 21.4% among HIV-infected pregnant women and was associated with higher plasma HIV levels [36]. This finding was notable: the prevalence of MG infection in the ART-naive participants was 6.4%, which was higher than that of CT or NG infection in the two groups. Therefore, we need further research to understand MG infection in the HIV-positive population in Yunnan Province.

We found that ART-naive individuals had a higher unemployment rate, unmarried rate, more sexual partners per year and less use of condoms than individuals receiving ART. Unemployment and a low income make marrying impractical in China, and individuals are more likely to proceed with unprotected sexual behavior for economic gains. However, individuals receiving ART recognized their condition and had more convenient access to STI preventative information. Our results show that the proportion of ever injecting drugs in the ART-naive group was lower than that in the ART group because sexual transmission has become the dominant route of HIV transmission. With the government’s strict control of drugs, intravenous drug use is rare. The average CD4^+^ T cell count, total T lymphocyte, count CD4/CD8 count, lymphocyte count and percentage of lymphocytes in the ART-naive participants were significantly lower than those in the participants receiving ART. In China, in 2015, the enrollment criteria for ART changed such that individuals should receive ART once they are diagnosed as HIV-positive. ART can inhibit the replication of HIV-1 and promote the gradual recovery of CD4^+^ T cell counts [37]. We found a significant promotion of CD4^+^ T cells and lymphocytes in the ART group. The participants receiving ART had higher average counts of white blood cells, platelets and triglycerides but a lower average erythrocyte count and trioxypurine level. Lipid abnormalities and metabolic complications associated with ART may explain this finding [38, 39].

Our findings revealed that females were more likely to have curable STIs (CT, NG, MG, HSV-2 or active syphilis), regardless of being in the ART-naive group or ART group. Two variables, female sex and ever injecting drugs, showed a significant association with CT–HIV coinfection. Similarly, female sex and an age > 36 years showed an association with HSV-2/HIV coinfection. Homosexual or bisexual status was associated with a significantly increased MG–HIV coinfection risk. However, an age between 18 and 35 years was a risk factor for TP–HIV or NG–HIV coinfection. Other risk factors for TP–HIV coinfection were homosexual or bisexual status, and NG–HIV coinfection or HIV/HBV coinfection. Participants receiving ART were less likely to have NG–HIV coinfection than those not receiving ART. In conclusion, the risk factors associated with sexually transmitted infections among HIV-positive individuals were female sex, an age between 18 and 35 years, ever injecting drugs, homosexual or bisexual status, HIV/HBV coinfection, and not receiving ART. A study revealed that multiple STIs were mainly found among MSM (14.8%) and bisexual individuals (23.5%), and MSM had the highest sex behavior-related risk for STIs in southern Italy [40].

Our results on STI–HIV coinfection have important implications for improving STI screening and treatment services among HIV-positive individuals. Evidence shows that many people with STIs are asymptomatic. The prevalence of syphilis was 13.5%; among the cases, 91.3% were asymptomatic cases among women in China. Most of the people with anorectal (93.3%) and urethral (79.2%) MG infections were asymptomatic among MSM in western Sydney [15]. A Nigerian study showed that over 95% of STI cases were asymptomatic among MSM [41]. In Yunnan, we found that some hospitals and clinics may not screen for other STIs when diagnosing a new HIV-positive patient, especially in resource-poor settings and rural areas, unless they have obvious symptoms. The symptom-based management of STIs is limited, and current and future trends are the laboratory diagnosis of STIs. Point-of-care testing and microfluidic and high-throughput omics technologies promise to revolutionize the diagnosis of STIs [42]. Therefore, strategies to screen all HIV-positive individuals for HSV-2, syphilis, MG, and so on would be necessary. Untreated STIs may facilitate HIV acquisition and transmission and reduce the effectiveness of antiviral therapy.

There are several limitations to the research. This was a cross-sectional study, and the participants were not random. Our collected information might have been compromised by recall bias and social desirability bias, as sexual behavior is private information to participants. Our recruitment was limited to one infectious hospital, and the regional diversity of the samples was not enough, which might influence the external validity of our results.

## Conclusions

In conclusion, we identified a high prevalence of STIs among ART-naive and HIV-positive individuals receiving ART in Yunnan. HSV-2, syphilis and MG were the most common STIs in HIV-positive individuals. HIV-positive individuals tend to neglect or hide their genital tract discomfort; thus, we suggest strengthening STI joint screening and treatment services among HIV-infected individuals regardless of whether they describe genital tract discomfort. The prevalence of MG was high in ART-naive participants, and further research is needed to understand this association. Younger patients, women, those who ever injected drugs, homosexual or bisexual individuals, those with coinfection with HBV and those not receiving ART should be a focus for interventions to decrease STIs.

## Data Availability

The majority of the datasets used and/or analyzed during the current study are available from the indicated published resources. The remaining data, including the model code, are available from the corresponding author on reasonable request.
